# Cestode parasite accumulation in *Octopus maya*: Insights from an opportunistic sampling during the 2022 red tide event

**DOI:** 10.1007/s00436-025-08560-7

**Published:** 2025-11-03

**Authors:** Linda Yacsiri G. Marmolejo-Guzmán, Jhonny G. García-Teh, Karen Ascenet Arjona-Cambranes, Guadalupe Anai May-Sosa, M. Leopoldina Aguirre-Macedo

**Affiliations:** 1 Department of Marine Resources, Center for Research and Advanced Studies, Campus Merida, Km 6 Carretera Antigua Progreso, Cordemex, 97319 Mérida, Yucatán México; 2 Instituto Tecnológico de Conkal, Km 16.3 Antigua Carretera Mérida-Motul, Conkal, Yucatán México

**Keywords:** *Octopus maya*, *Prochristianella* sp., Host size relationship, Sampling event, Small octopuses

## Abstract

The infection dynamics of *Prochristianella* sp., a metacestode parasitizing the Yucatán Peninsula-endemic octopus, *Octopus maya*, were examined to explore the relationship between host size and parasitic infection parameters. During a red tide event, forty-nine octopuses were sampled from a single locality in the Yucatán Peninsula, México, and classified into three size classes. Infection metrics were assessed, including prevalence, mean intensity, abundance, and total parasite count. The results revealed a significant positive association (r = 0.85) between host size and the number of *Prochristianella* sp., with larger octopuses exhibiting higher infection parameters. The findings suggest that *Prochristianella* sp. progressively accumulates in *O. maya* throughout its very early life, aligning with the general patterns observed in host-parasite interactions. This finding is consistent with previous studies, which suggest that larger hosts may accumulate higher parasite loads due to their prolonged exposure to infective stages and their trophic habits. These results highlight the ecological role of *O. maya* as an intermediate host in its marine ecosystem, underscoring the potential implications of parasitic infections on its health and population dynamics. This study represents a significant step toward understanding the ecology of parasites exploiting *O. maya*, providing insights into host-parasite relationships in marine cephalopods and offering a foundation for future research on the health and sustainability of this economically important species.

## Introduction

Endemic to the Yucatán Peninsula, México, *Octopus maya* is one of the most ecologically and economically important species in the region. It plays a crucial role in the marine ecosystem, sustaining the local fishing industry. Indeed, it is estimated to contribute over 60% of the total octopus catch (Avendaño et al. 2019; Coronado et al. 2020). The Yucatán octopus fishery is the largest in the Americas and is regulated by the National Fisheries Charter and Mexican Official Standards NOM-008-SAG/PESC-2015 (2015a) and NOM-009-SAG/PESC-2015 (2015b). These regulations ensure sustainable practices by setting a minimum capture size of 110 mm mantle length, prohibiting the use of hooks and spears, and enforcing a closed season from December 16 to July 31 (Rosas et al. 2014; Guarneros-Narváez et al. (2016). However, these regulations also limit scientific research opportunities, especially for collecting specimens under regulated sizes. The parasite fauna of *O. maya* comprises 20 parasite taxa, including seven cestode species, with *Prochristianella* sp. exhibiting the highest prevalence and abundance, affecting the octopus's health by causing tissue alterations and fibrosis in the anterior salivary glands (Guillén-Hernández et al. 2018a, b; Marmolejo-Guzmán et al. 2022).

Harmful algal blooms (HABs), or red tides, are spontaneous natural phenomena that appear on the coasts and significantly affect the marine ecosystem; their duration varies from days to months; their appearance is not limited to one time of the year; however, they are more common in summer (Rodríguez-Gil et al. 2007). These events result from the massive growth of phytoplankton due to favorable marine conditions, such as nutrient availability and specific currents and winds (Aguilar-Medrano et al. 2023; Hallegraeff 2023). Recently, non-toxic but harmful algal blooms have been observed along the Yucatán coast, causing significant fish and marine organism deaths (Rodríguez-Gil et al. 2007; Zetina-Ríos et al. 2009). The 2022 red tide, dominated by the dinoflagellate *Cylindrotheca closterium*, was a notable ecological disturbance that began at the port of San Felipe and progressed westward to Chelem, lasting approximately a week and resulting in widespread mortality of marine organisms, including specimens of *O. maya* (Aguilar-Medrano et al. 2023).

The 2022 red tide event offered an opportunity for *O. maya* sampling across a broad size range, overcoming the restrictions of Mexican Official Standards (NOM). This event resulted in numerous moribund and freshly deceased octopuses washing ashore, allowing us to collect specimens that span different developmental stages. This unprecedented sampling opportunity enabled a comprehensive study on the progression of infection of Trypanorhyncha cestodes (marine tapeworms with elasmobranch definitive hosts) in *O. maya*. By examining octopuses of a size gradient, we gain valuable insights into the prevalence, intensity, and pathological effects of these infections across different life stages of *O. maya*. These baseline data contribute to the ecological understanding of octopus-parasite systems, though direct fishery impacts remain unquantified.

## Materials and methods

### Specimen recollection

**Fig. 1:**
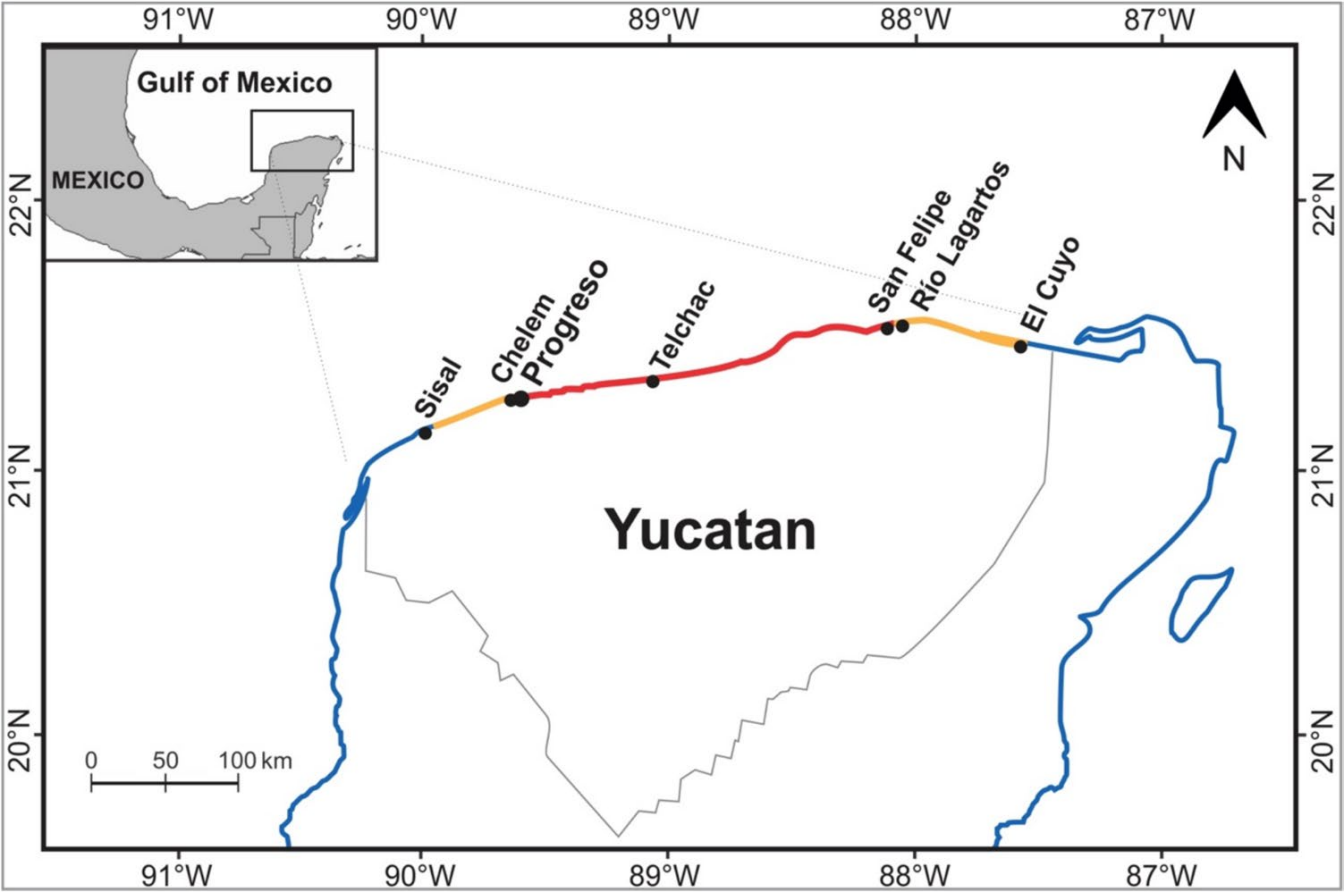
Main fishing ports in Yucatán affected by the 2022 red tide event. The red line represents the highly affected area. The orange line represents the medium-affected area. Map created by the authors, based on COBI (2022)

During the "red tide" phenomenon on the coasts of the state of Yucatán in August 2022, 34 moribund and visibly fresh dead specimens of the mayan octopus (*O. maya*) were recollected from the coasts of the municipality of Progreso, Yucatán, México (Fig. 1). The specimens were transported in cool boxes to the Aquatic Pathology Laboratory at Cinvestav Mérida, where they were preserved in freezers at −20 °C for subsequent examinations. Once in the laboratory, morphometric data were collected for each specimen, including dorsal mantle length (DML) in millimeters and weight in grams.

### Helminthological examination

**Fig. 2:**
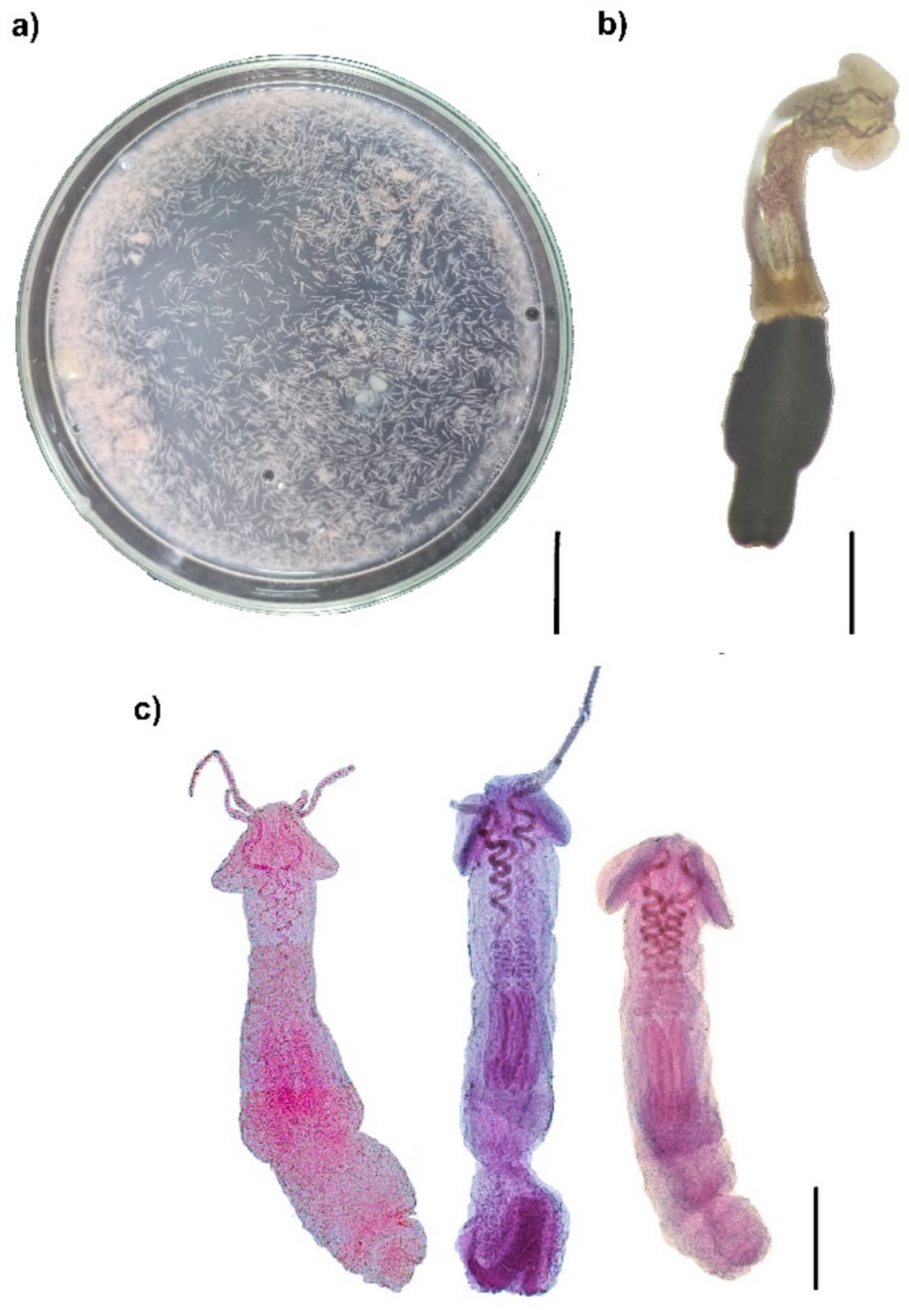
Morphological confirmation of *Prochristianella* sp. cestodes. **a)** A Petri dish with *Prochristianella* sp. individual extracted from a single octopus, **b)** a detailed view of a single *Prochristianella* sp., **c)** individuals of *Prochristianella* sp. at different developmental stages within the plerocercoid phase. Bar scale: **a)** 2000 µm, **b)** 200 µm, and **c)** 200 µm

For the helminthological examination, the octopuses were dissected. Their organs (buccal mass, renal sacs, ink sac, stomach, siphon, spiral cecum, crop, heart, and gills) were examined on glass slides under a stereoscopic microscope (Motic SMZ-168) (Fig. 2a). *Prochristianella* sp. plerocercoids were encapsulated in a single structure (presumably elicited as a host response to *Prochristianella* sp. infection), primarily on the superior mandibular muscle of the buccal mass. At the same time, some free individuals were found in other digestive system organs (Marmolejo-Guzmán et al. unpublished data). All *Prochristianella* sp. individuals were separated, counted, and used for further analysis in this study.

Some *Prochristianella* sp. individuals were fixed in vials with 4% formalin for staining purposes (others were preserved differently for additional analyses or discarded). From these, five randomly selected specimens per octopus (n = 34 hosts; total 170 individuals) were stained with Mayer-Schuberg's carmine technique, mounted in Canada balsam (Palm 2004), and identified as *Prochristianella* sp. according to Palm (2004) (Fig. 2b). All collected individuals were confirmed as *Prochristianella* sp. despite the varied developmental stages of the plerocercoid phase (Fig. 2c). The best-preserved specimens were deposited in the Helminthological Collection of Cinvestav Unit Mérida (CHCM) with accession codes CHCM-705, CHCM-705.2, and CHCM-705.3.

### Size class categorization

Dorsal mantle length (DML) was measured for all specimens, with data presented as mean ± standard deviation (SD) for each size class. *Octopus maya* specimens were categorized into three size classes based on dorsal mantle length (DML): small (< 50 mm), medium (51–100 mm), and large (101–150 mm). The small size class corresponds to juvenile individuals, consistent with the developmental definition proposed by Rosas et al. (2014) beginning at 14 days post-hatching (DPH) and supported by wild growth rates of 0.425 mm/day (Solís and Chávez 1986), indicating these sizes represent immature individuals below sexual maturity thresholds (450 g ≈ 110 mm DML) (Rosas et al. 2014). Medium and large classes represent progressively mature stages, where the 110 mm DML minimum catch size (NOM-008-SAG/PESC-2015 2015a) aligns with both the modal size in wild populations (Nepita Villanueva and Defeo 2001) and documented reproductive maturity (Rosas et al. 2014).

Due to the sample limitation, only two of the 34 individuals collected during the red tide event belonged to the largest size category (101–150 mm DML). Consequently, morphometric and parasitological data of 15 additional *O. maya* specimens in the largest category from the fishing season of 2021 (August–December) were incorporated for the analyses. This inclusion was intended to enhance the representation of larger specimens.

### Data analysis

To analyze the progression of *Prochristianella* sp. infections, the prevalence and abundance were calculated following the recommendations of Bush et al. (1997). Data on the prevalence and abundance of cestode infections across different classes of *O. maya* were statistically analyzed to understand the infection dynamics.

A nonparametric Kruskal–Wallis rank sum test was applied to detect differences between host size classes and the number of *Prochristianella* sp. individuals. Subsequently, the Dunn post-hoc test with Holm's adjustment method was applied to account for multiple comparisons. Additionally, Spearman's rank correlation was used to determine a possible significant association between octopus size (DML) (independent variable) and the number of *Prochristianella* sp. individuals (dependent variable). Finally, the coefficient of variation was calculated to determine the percentage of the total variation in the number of *Prochristianella* sp. individuals that was due to variation in octopus DML size (Sokal and Rohlf 1995). All statistical analyses were performed in R (R Development Core Team, http://www.R-project-com) using cort.test function and library "dunn.test". Statistical significance was set at α < 0.05 unless otherwise stated.

## Results

**Fig. 3:**
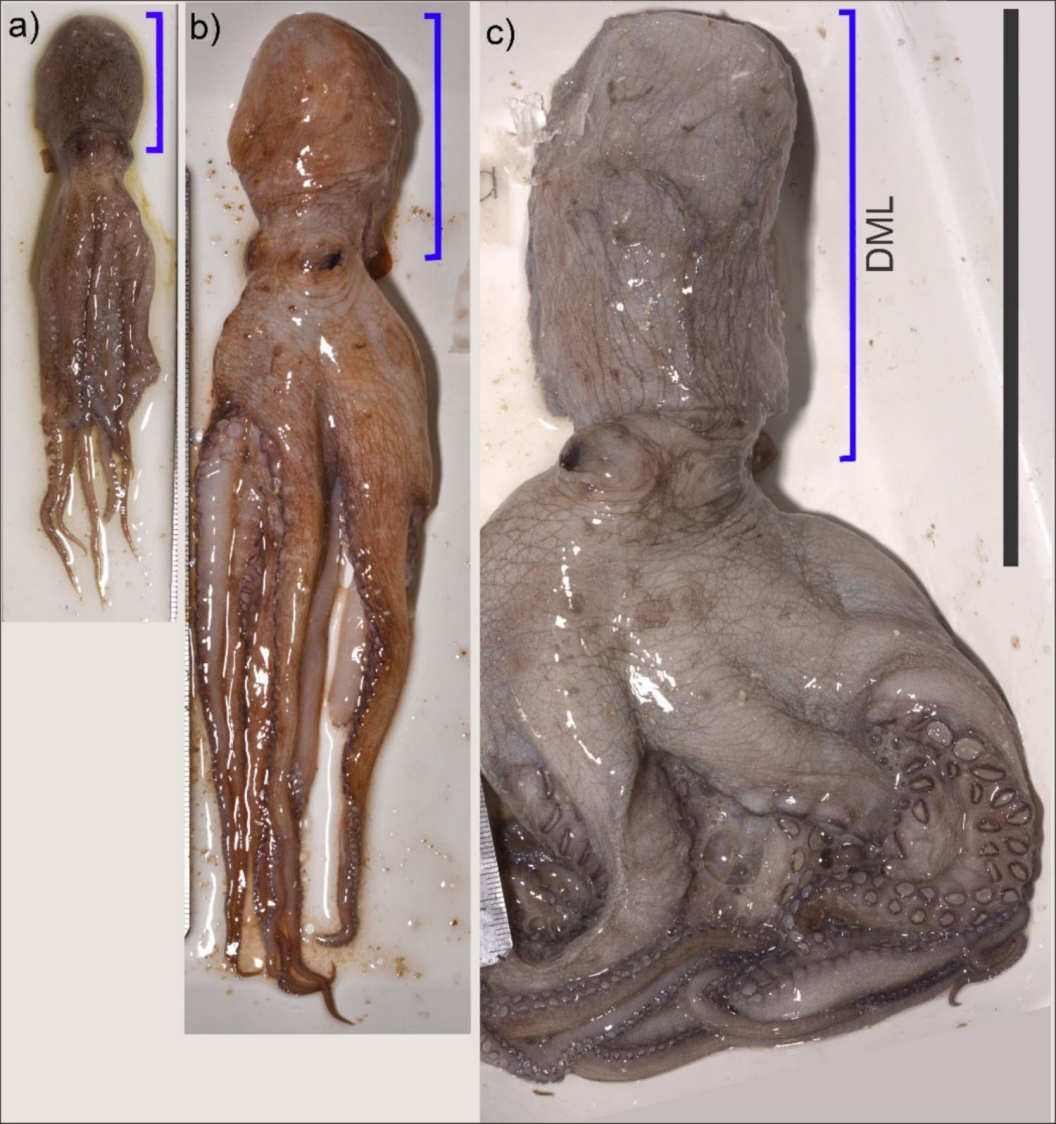
Specimens of *Octopus maya* collected during the 2022 red tide event in Yucatán, México. **a**) Small size specimen; **b**) Medium size specimen; c) Large size specimen. Scale bar = 110 mm; blue line = Dorsal Mantle Length (DML)

**Fig. 4:**
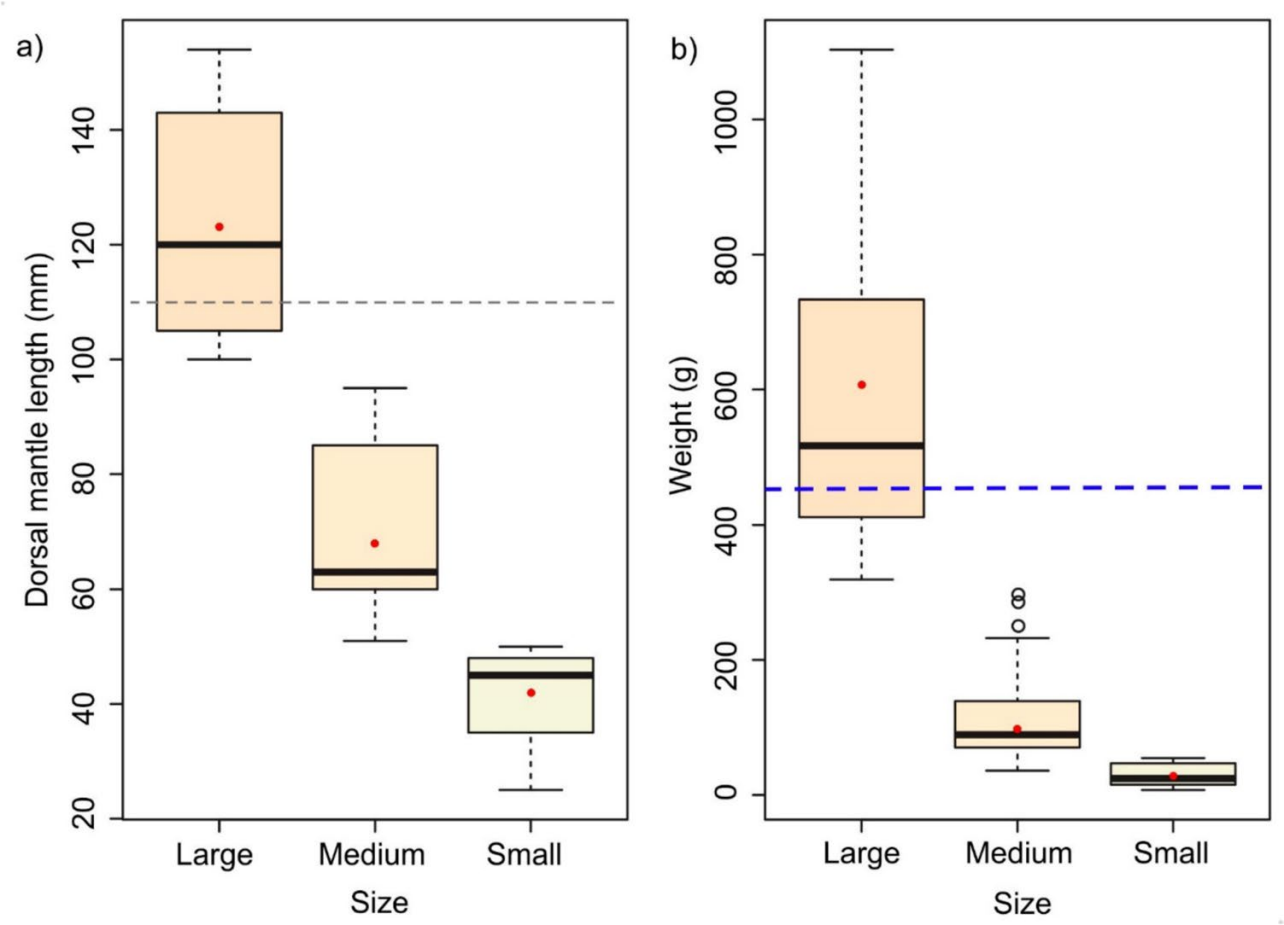
Dorsal mantle length (**a**) and body weight (**b**) of *Octopus maya* across size classes. The red circle represents the mean values. Dashed lines indicate reference thresholds: grey for the minimum legal catch size (110 mm DML, NOM-008-SAG/PESC-2015 2015a), and blue for the average weight at sexual maturity (450 g, NOM-008-SAG/PESC-2015 2015a; Rosas et al. 2014)

Forty-nine specimens of *O. maya* were examined (Fig. 3). The dorsal mantle length (DML) ranged from 25 to 154 mm, with a mean of 81.1 ± 36.9 mm, and weight from 7.2 g to 1103.4 g, with a mean of 269.7 ± 298.1 g (Table 1). The dorsal mantle length and weights by size classes are presented in Fig. 4.

**Table 1:** Infection parameters of *Prochristianella* sp. in *Octopus maya* are categorized by size classes based on dorsal mantle length (DML) from Progreso, Yucatán Peninsula

Parameters	Overall	Small	Medium	Large
	(49)	(13)	(20)	(16)
Range DML (mm)	25–154	25–50	51–100	102–154
DML (mm) $\overline{X }$ ±SD	81.1 ± 36.9	41.1 ± 8.4	71.9 ± 16.7	125.1 ± 19.7
Weight (g) ± SD	269.7 ± 298.1	29.1 ± 16.7	145.3 ± 103.7	620.8 ± 261.1
Prevalence (%)	96	85	100	100
Abundance $\overline{X }$ ±SD	237 ± 222	39 ± 38	182 ± 180	467 ± 144
Intensity	1–812	2–97	1–585	316–812
No. *Prochristianella* sp.	11,619	509	3635	7475

$\overline{X }$ -mean; SD-standard deviation; %-percentage; the numbers in parentheses correspond to the octopus examined

While other cestodes (Onchoproteocephalidae and Trypanorhyncha species) were registered during the parasitological examination, this study focused exclusively on *Prochristianella* sp. All examined cestodes of this genus were confirmed to belong to *Prochristianella* sp., through morphological analysis (Fig. 2c), consistently identified across all size classes and developmental stages. While previous molecular analysis (28S rDNA) showed genetic similarity to *Prochristianella* sp. 1 (GenBank: DQ642769) (Marmolejo-Guzmán et al. 2022), formal morphological identification (particularly of tentacle armature patterns following Palm 2004) could not be completed due to suboptimal preservation conditions of the cestode specimens. We therefore cautiously designate these larvae as *Prochristianella* sp. (= *Prochristianella* sp. 1 from Marmolejo-Guzmán et al. 2022) until comprehensive morphological and molecular characterization can be performed.

*Prochristianella* sp. plerocercoid reached 96% prevalence, with an average abundance of 237 ± 222 individuals per examined *O. maya*. In total, 11,619 *Prochristianella* sp. plerocercoid individuals were recorded in this parasitological study (Table 1).

Infection progression was analyzed across the three previously defined octopus size classes: 13 specimens were classified in the small-size class (< 50 mm DML), 20 in the medium-size class (51–100 mm DML), and 16 in the large-size class (101–150 mm DML). A prevalence of 85% *Prochristianella* sp. infection was observed for the small-sized octopus group, with a mean abundance of 39 ± 38 individuals and an infection intensity ranging from 2 to 97 cestodes per infected host (Table 1). The medium-sized class exhibited a 100% prevalence, with a mean abundance of 182 ± 180 cestodes per octopus and an infection intensity ranging from one to 585 cestodes per infected host (Table 1). The large-sized class individuals were the most heavily infected, with a 100% prevalence, the highest mean abundance (467 ± 144 cestodes per octopus), and infection intensities ranging from 316 to 812 cestodes per infected host (Table 1).

**Fig. 5:**
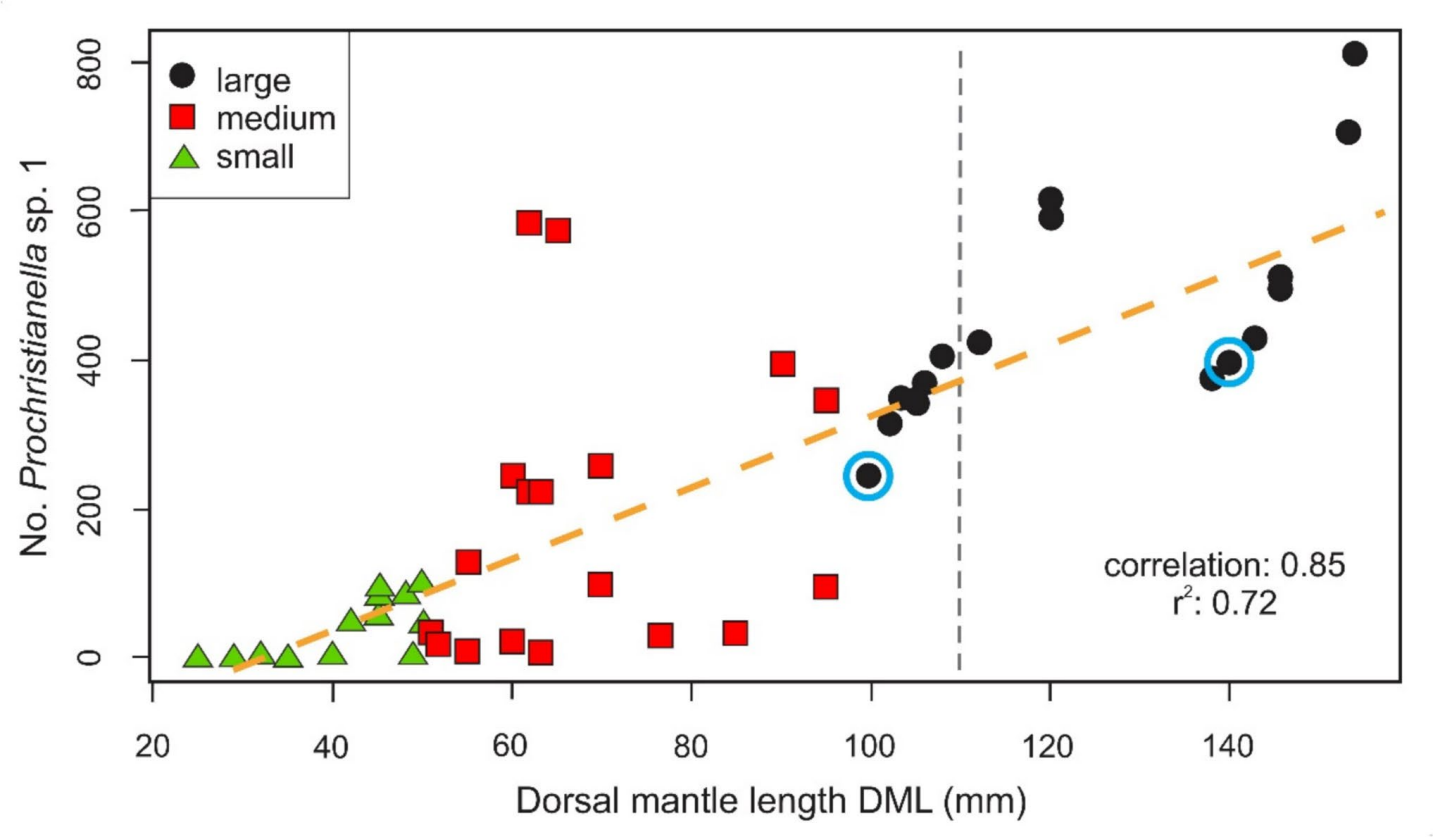
Relationship between *Prochristianella* sp*.* abundance and *Octopus maya* size classes. Significant differences in parasite abundance were observed among the three octopus size groups (Kruskal–Wallis, H (2,47) = 28.55; p = 6.31e-07). A strong positive correlation (r = 0.85) was identified, with octopus size explaining 72% of the variation in *Prochristianella* sp*.* numbers. The vertical dashed line (grey) represents the minimum catch size allowed (110 mm). The blue circle corresponds to the only large-sized individuals collected during the red tide

Statistically significant differences were found between octopus size classes and the number of *Prochristianella* sp. individuals (Kruskal–Wallis, *H*_(2,46)_ = 29.02; *p* = 4.98e-07). Dunn's multiple comparison post-hoc test revealed significant differences among all size classes (p < 0.05 for the comparisons). Additionally, a positive association (r = 0.85) was found between octopus size and the number of *Prochristianella* sp. individuals. Finally, it was found that 72% of the variation in the number of *Prochristianella* sp. can be attributed to changes in the octopus size (Fig. 5).

## Discussion

The 2022 red tide on the north coast of the Yucatán Peninsula provided a valuable opportunity to investigate the progression of *Prochristianella* sp. infection during the early developmental stages of *O. maya*. For the first time, individuals measuring below the minimum legal capture size (110 mm) established by the Mexican Standard NOM-008-SAG/PESC-2015 were available for parasitological examination. The analysis of 34 specimens of *O. maya*, classified as small (< 50 mm), medium (51–100 mm), and large (101–150 mm), confirmed the infection of *Prochristianella* sp. at an early octopus stage, with a DML of 25 mm and a weight of 7.2 g. These results agree with those of Guillén-Hernández et al. (2018b, 2024) and Marmolejo-Guzmán et al. (2022), who found that *Prochristianella* sp. exhibited the highest cestode prevalence and abundance in *O. maya,* the octopus.

These results suggest that the life cycle dynamics and ecological interactions of *Prochristianella* sp. are particularly well-suited to its host, potentially enabling its accumulation and persistence throughout the octopus's life span. At the same time, the presence of highly infected hosts from the early life octopus stage (> 90%) highlights its role as a core parasite species of *O. maya* (Holmes 1991).

The progression of *Prochristianella* sp. infection in *O. maya* observed in this study aligns closely with the general characteristics of macroparasite infections described in the literature (Delahay et al. 2009; Guillén-Hernández et al. 2024). Macroparasites are known to induce chronic infections with short-lived or negligible immunity, which often facilitates re-infection in their hosts (Delahay et al. 2009).

Therefore, larger octopuses may accumulate higher parasite loads through ecological mechanisms including, 1) prolonged exposure to infected prey through trophic transmission (Guillén-Hernández et al. 2018a), 2) increased consumption of intermediate/paratenic host with age, and 3) seasonal fluctuations in host availability driven by temperature and photoperiod in tropical benthic systems (Coma et al. 2000). Complementing the work of Guillén-Hernández et al. (2024), who reported size-dependent abundance of *Prochristianella* sp. in *O. maya* across multiple life stages, including examination of gonadal development effects, this study specifically analyzes infection patterns in smaller octopuses (< 50 mm). These size-related differences in parasite load (< 50 mm) may suggest early-life exposure risks, possibly involving trophic pathways. However, conclusions remain limited by unconfirmed transmission mechanisms due to the unknown life cycle of *Prochristianella* sp. and the lack of physiological data in this study.

The strong positive relationship between *O. maya* size and *Prochristianella* sp. abundance (r^2^ = 0.72; p < 0.001) reflects progressive accumulation through the trophic transmission pathway mediated by host feeding ecology. Lower infection rates in smaller individuals (0–50 mm DML) likely reflect a reduced consumption of intermediate hosts, potentially due to size-limited prey selection or differing prey preferences compared to adults (Markaida 2023; Guillén-Hernández et al. 2024). As an intermediate consumer, *O. maya* acquires parasites through the ingestion of diverse prey, with larger hosts encountering more infected prey through ontogenetic dietary expansion**.** While crustaceans like *Pitho* spp. dominate the diet of adult *O. maya* in Campeche (Markaida 2023), the specific intermediate host responsible for *Prochristianella* sp. transmission remains unidentified, particularly in Yucatán populations where dietary studies are lacking. Aside from the fact that the host transmitting *Prochristianella* sp. to the octopus has not been identified, the result suggests that such an intermediate host may be an octopus regular prey item throughout its entire life.

Helminths such as cestodes and larval digeneans often exhibit a positive relationship with host size due to trophic transmission, as larger hosts consume more diverse and abundant prey (Price and Clancy 1983). This pattern is supported by meta-analyses showing that fish length correlates significantly with infection intensity in trophically acquired parasites such as cestodes and digeneans (Poulin 2000). However, effect sizes vary across species and systems. For instance, in *Chromis cyanea*, the relationship between host size and parasite load explains a broad range of variance (r^2^ = 0.11–0.72) (Fernández-Osorio et al. 2015). These parallels highlight that prolonged exposure and expanding habitat use primarily explain infection patterns across taxa (Sánchez et al. 2018), reinforcing the need for size-structured studies of trophic transmission.

The 2022 red tide mortality event enabled the first parasitological assessment of small *Octopus maya* below legal harvest size (110 mm DML), complementing previous work on adult infections (Guillén-Hernández et al. 2024). The strong positive correlation between host size and parasite abundance (r^2^ = 0.72) confirmed a progressive accumulation, most likely through both continuous exposure and increased consumption of infected prey, facilitated by expanded habitat use in larger octopuses. These findings establish an ecological baseline for monitoring potential parasite-mediated population effects under future environmental changes. Documenting infection dynamics across ontogeny may prove helpful if parasitism interacts with emerging stressors, such as shifting prey availability or habitat alterations. On the other hand, the effect of red tides on parasite transmission would require determining their impact on the availability of the first intermediate host, which can only be studied through a time series when another red tide occurs.

## Data Availability

Datasets generated and analyzed are available through the corresponding author.
